# Supplementary material for: RhoGDI2-Mediated Rac1 Recruitment to Filamin A Enhances Rac1 Activity and Promotes Invasive Abilities of Gastric Cancer Cells
Source: Cancers (Basel). 2022 Jan 5;14(1):255. doi: 10.3390/cancers14010255 (PMC8750349; doi:10.3390/cancers14010255)
Supplement: Supplementary file 1 [file cancers-14-00255-s001.zip › cancers-1492233-supplementary.pdf]

Supplementary materials: RhoGDI2-Mediated Rac1 Recruitment to Filamin A Enhances Rac1 Activity and Promotes Invasive Abilities of Gastric Cancer Cells

Hyo-Jin Kim, Ki-Jun Ryu, Minju Kim, Taeyoung Kim, Seon-Hee Kim, Hyeontak Han, Hyemin Kim, Keun-Seok Hong, Chae Yeong Song, Yeonga Choi, Cheol Hwangbo, Kwang Dong Kim and Jiyun Yoo

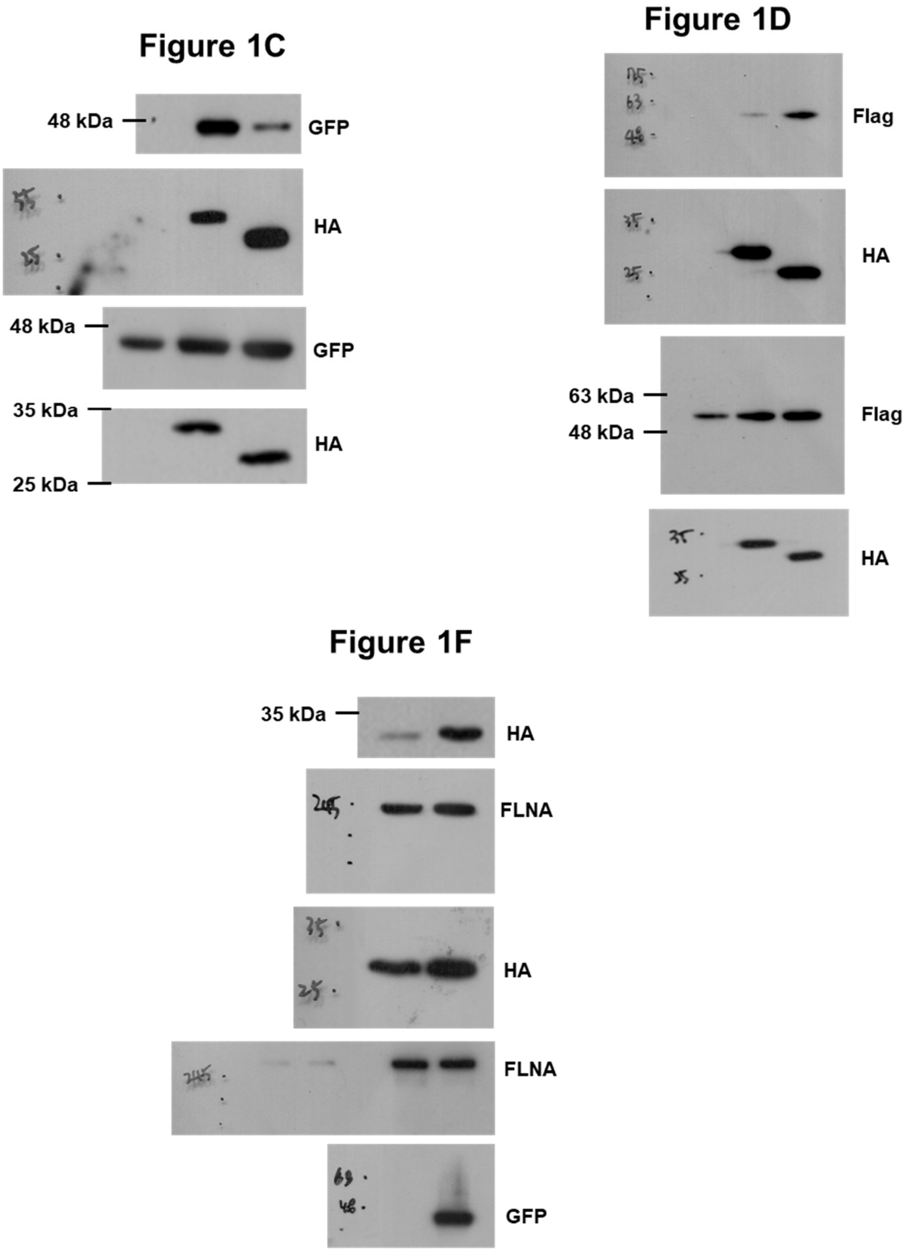

Figure S1. Raw data of immunoblot from Figure 1C, 1D and 1F.

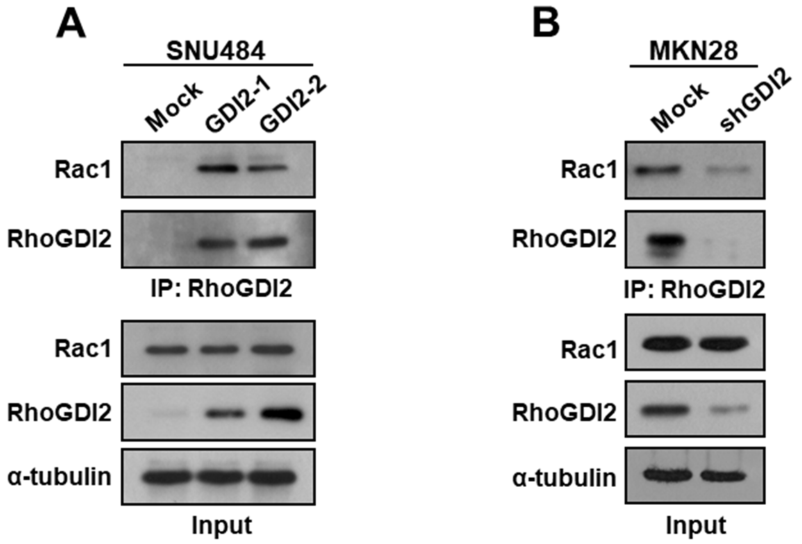

Figure S2. Interaction between RhoGDI2 and Rac1 in RhoGDI2-overexpressing SNU484 and RhoGDI2-depleted MKN28 cells. RhoGDI2-overexpressing SNU484 (GDI2-1 and GDI2-2) cells (**A**) and RhoGDI2-depleted MKN28 cells (**B**) were immunoprecipitated with anti-RhoGDI2 antibody and analyzed by western blot with anti-Rac1 antibody.

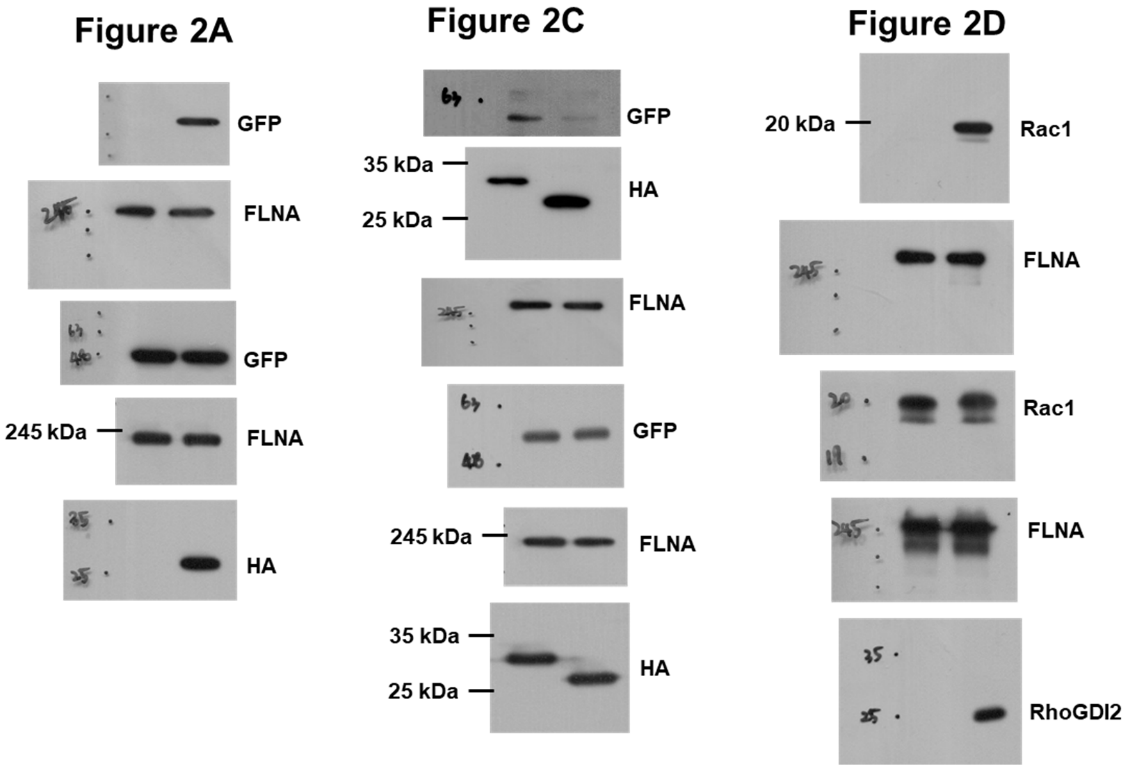

Figure S3. Raw data of immunoblot from Figure 2A, 2C and 2D.

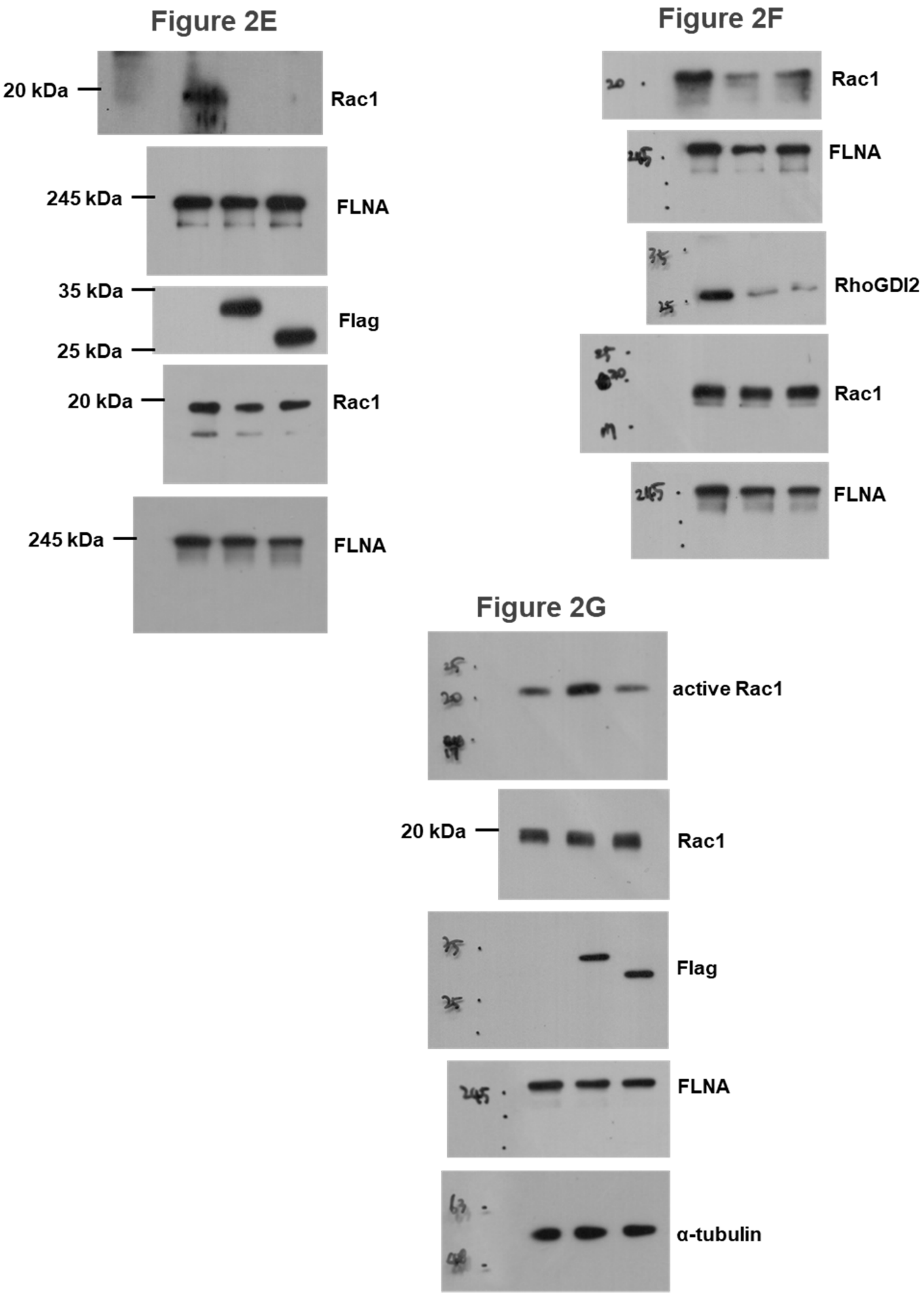

Figure S4. Raw data of immunoblot from Figure 2E, 2F and 2G.

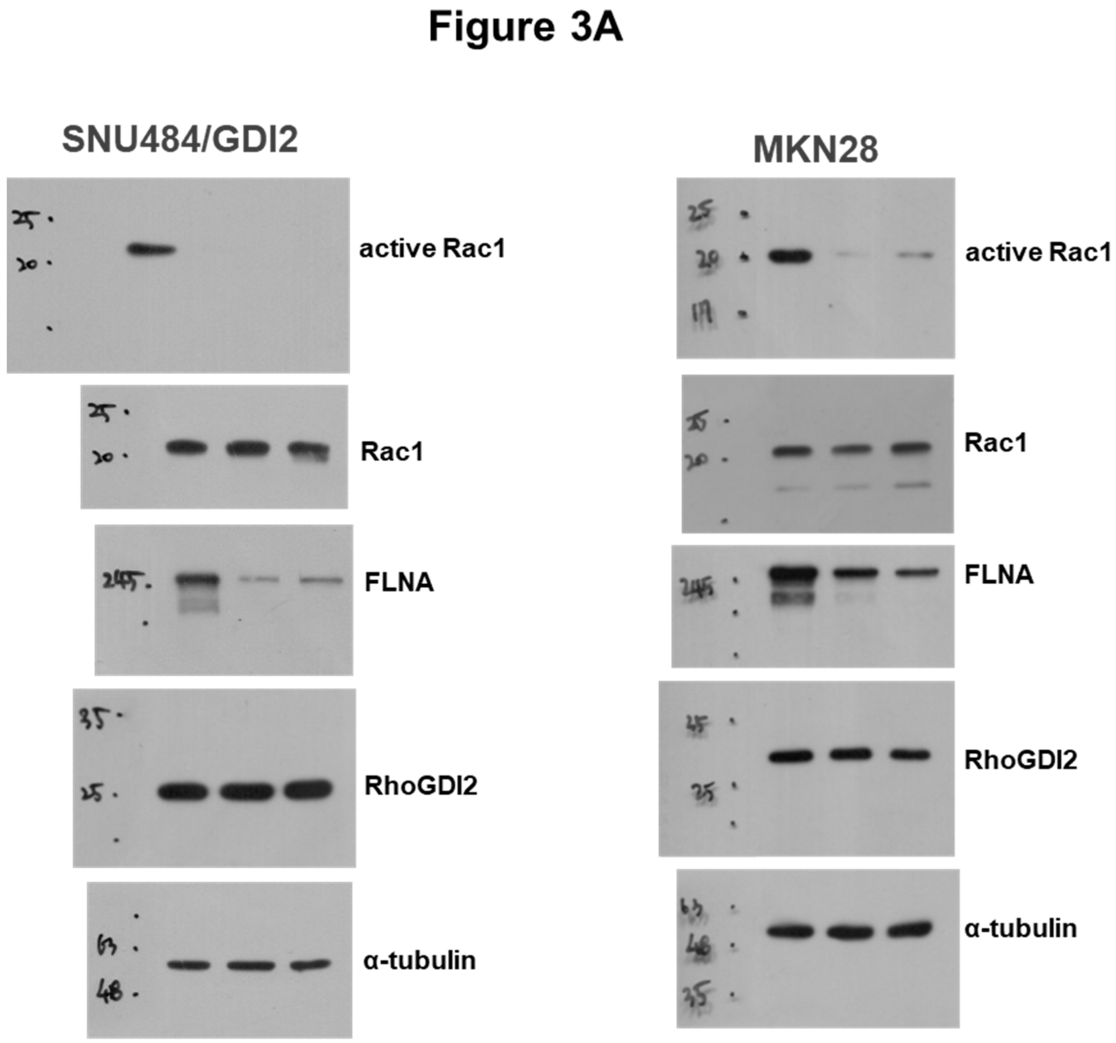

Figure S5. Raw data of immunoblot from Figure 3A.

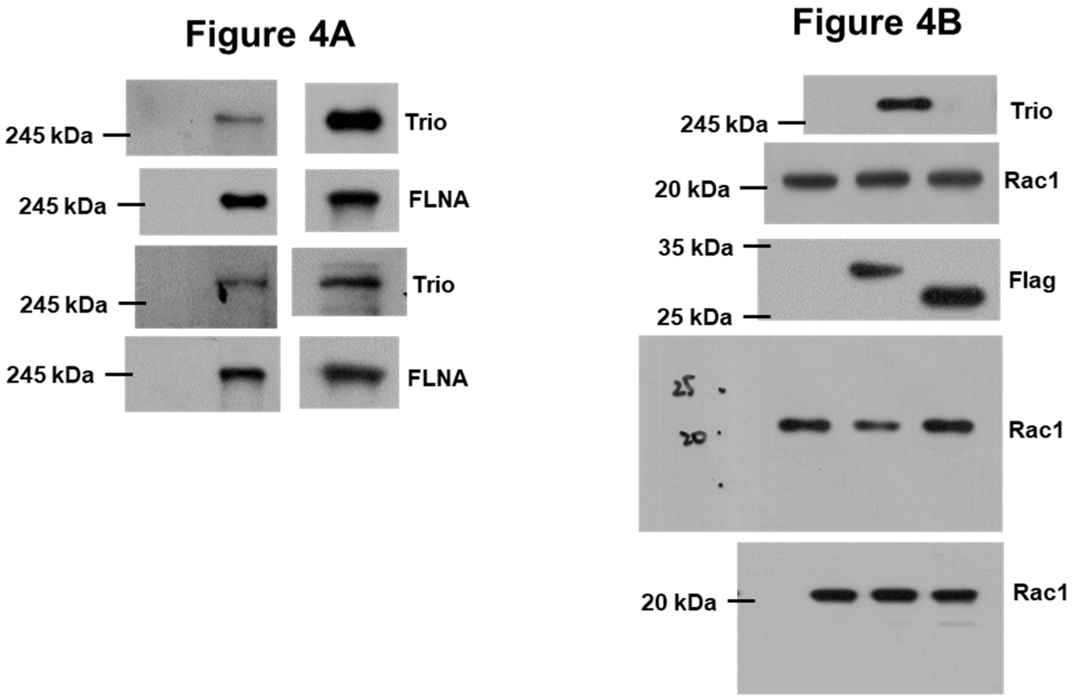

Figure S6. Raw data of immunoblot from Figure 4A and 4B.

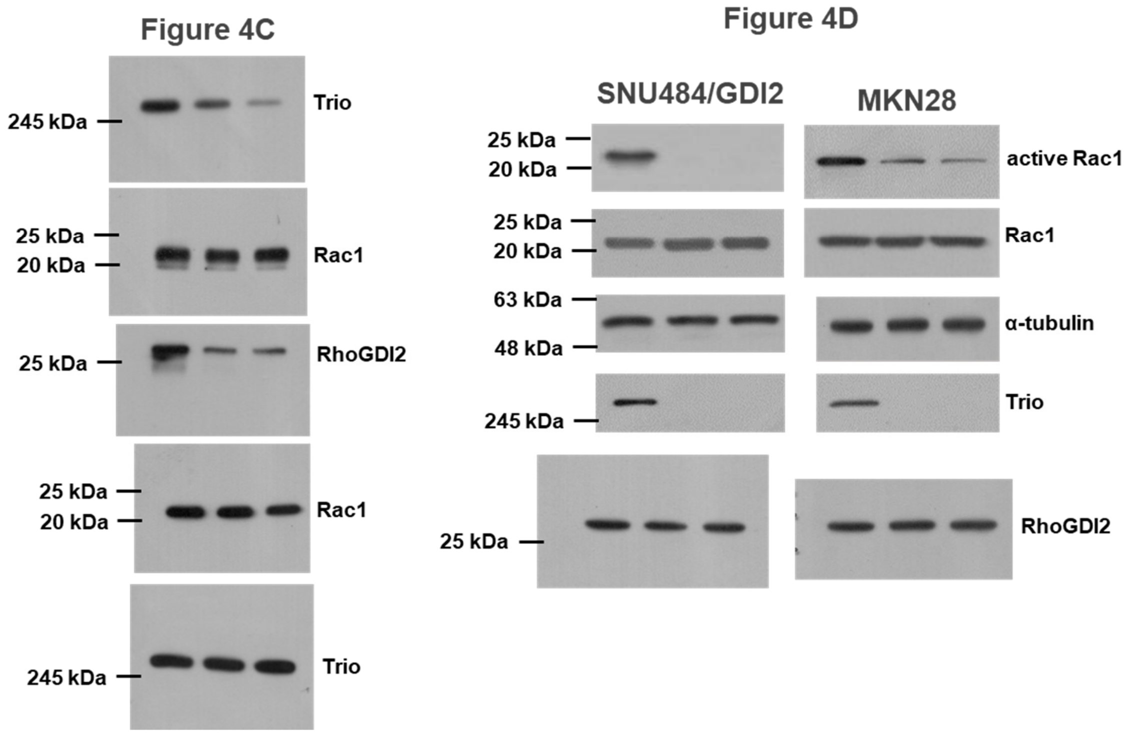

Figure S7. Raw data of immunoblot from Figure 4C and 4D.
